# Further phenotypic delineation of subtelomeric (terminal) 4q deletion with emphasis on intracranial and reproductive anatomy

**DOI:** 10.1186/1750-1172-2-9

**Published:** 2007-02-12

**Authors:** Eric Scott Sills, MJ Burns, Laurinda D Parker, Lisa P Carroll, Lisa L Kephart, CS Dyer, Peter R Papenhausen, Jessica G Davis

**Affiliations:** 1 Reproductive Medicine Associates at Vassar Brothers, Fishkill, New York, USA; 2 Murphy Women's Center, Murphy, North Carolina, USA; 3 Cytogenetics Laboratory, Laboratory Corporation of America, Research Triangle Park, North Carolina, USA; 4 Division of Human Genetics, Department of Pediatrics, Weill Medical College, Cornell University, New York, New York, USA

## Abstract

**Objective:**

To describe selected morphological and developmental features associated with subtelomeric deletion at chromosome 4q.

**Materials and methods:**

A 21-year old female was brought for gynecologic evaluation of menorrhagia. High-resolution metaphase karyotype and subtelomere fluorescent in-situ hybridization (FISH) analysis were used for genotype determination. Pelvic anatomy was characterized via CT and laparoscopy; MR and CT were used for intracranial imaging.

**Results:**

A *de novo *deletion [46,XX del(4)(q32)] was identified cytogenetically and confirmed as a terminal loss via subtelomere FISH. Hand/foot malformation characteristic of deletion at this segment was present. Pelvic CT and laparoscopy revealed normal uterine anatomy. Fallopian tubes appeared grossly unremarkable, and a right ovarian cyst was excised without difficulty. Bilateral broad ligament fibroadipose nodularities were noted adjacent to the uterus between round ligament and fallopian tube. Neurological exam revealed no focal defects, although brain MR identified an abnormal signal intensity at the inferior margin of the globus pallidus, consistent with old lacunar infarct and gliosis. Developmental delay was supported by an observed level of general intellectual function estimated at age seven.

**Conclusion:**

Terminal deletion of the long arm of chromosome 4 is a rare genetic event associated with a distinctive phenotype dependent on the size of the deletion. Chromosomal losses that span the 4q32 band include mental retardation and mild craniofacial anomalies. Here, further characterization of this disorder is offered including precise quantification of the DNA loss, information on brain morphology and pelvic anatomy. Additional studies will be required to characterize the full developmental and physiologic implications of this unusual genetic disorder.

## Background

The incidence of terminal deletion of chromosome 4q is believed to be very low but is not known with certainty. Indeed only a limited number of reports describing such deletions exist [1] and very few of these have specifically addressed reproductive and intracranial morphology in affected individuals. Here we present an analysis of the impact of this unusual autosomal deletion on pelvic and brain anatomy.

## Materials and methods

A 21 year-old Caucasian female nonsmoker G_0 _was brought by family for gynecologic evaluation to assess irregular menses and menorrhagia. She was fully ambulatory but from an early age demonstrated developmental delay; she resided in an institutional setting for several months prior to presentation. The patient was in good general health and had no specific medical complaint except occasional headache and irregular vaginal bleeding.

Review of neonatal records described coarse facial features, temporal narrowing, anteverted nose, posteriorly rotated ears, broad philtrum, hypoplastic left fifth digit, and 3^rd ^toe overriding 4^th ^toe (bilaterally). Although inborn errors of metabolism were considered, the diagnosis of "deletion of the long arm of the fourth chromosome" was made a few days after delivery. The parents underwent testing and normal karyotypes were confirmed for both. The patient underwent an uncomplicated right inguinal hernia repair at age 4 months, but there was no other surgery.

Although no recent formal developmental testing had been performed, careful history provided by the patient's caregivers revealed that by age 21 the patient had a level of basic cognitive function normally seen in a 7-year old child. Specifically, the patient was able to attend to her own toilet needs and even follow simple instructions to prepare meals. She knew the alphabet and could correctly identify numerals but was unable to read or write, except printing her own name. Arithmetic was not possible. Primary colors were recognized but the patient had no concept of interpreting calendars, clocks, or counting money. No particular affinity to music or other artistic expression was noted. There was never any hearing or visual impairment and the patient mastered good communication skills.

From our center, genotype was confirmed by karyotype of PHA-stimulated peripheral metaphase leukocytes (*n *= 5) by GTG banding at 550 cM resolution. Subtelomere FISH was performed with TelVysion Probes (Vysis/Abbott Molecular, Des Plains, IL). Denaturation of metaphase chromosomes, hybridization, and washing was according to manufacturers recommendations. After DAPI counterstaining, microscopy and photography was performed under epifluorescence.

Liquid-based Papanicolau test was performed for cervical cytology. Pelvic anatomy was evaluated by transvaginal ultrasound and CT. When an echolucent 45 mm right ovarian cyst was identified on ultrasound, surgery was planned for excision. Triple-puncture 5 mm laparoscopy was undertaken as described previously [2].

Using a 1.5 Tesla instrument, intracranial anatomy was elucidated via axial T1, T2, FLAIR DW1 and ADC magnetic resonance images with T1 sagittal pulse sequence including post-contrast axial/coronal T1-weighted sequences. Standard radiographs of both hands were also obtained.

## Results

**Figure 1:**
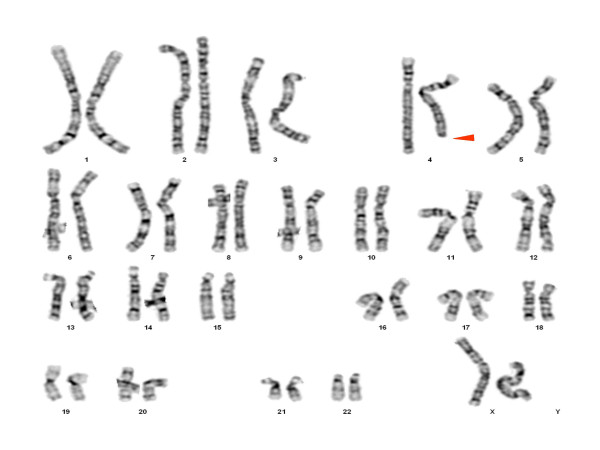
Peripheral GTG banded metaphase karyotype for 46,XX del(4)(q32) proband indicating region of deleted chromosome 4 (arrow).

**Figure 2:**
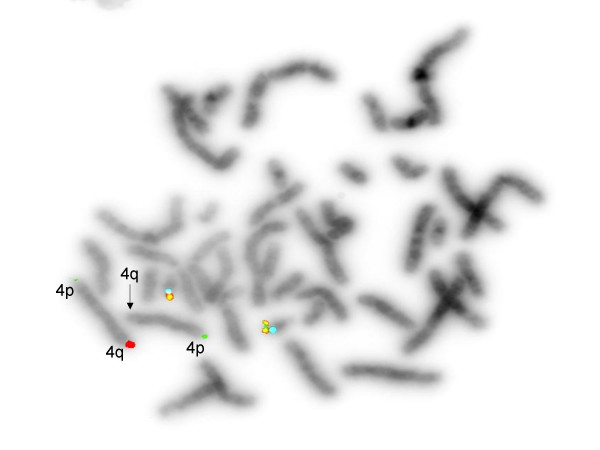
Fluorescent in situ hybridization study indicating deletion of distal long arm of chromosome 4. (Green = 4p, Red = 4q [unpaired signal]; chromosome 21 = control).

Genetic assessment showed 46,XX, del(4)32q [Figure 1]; a terminal deletion was confirmed by FISH analysis [Figure 2]. Both parents had a normal karyotype.

**Figure 3:**
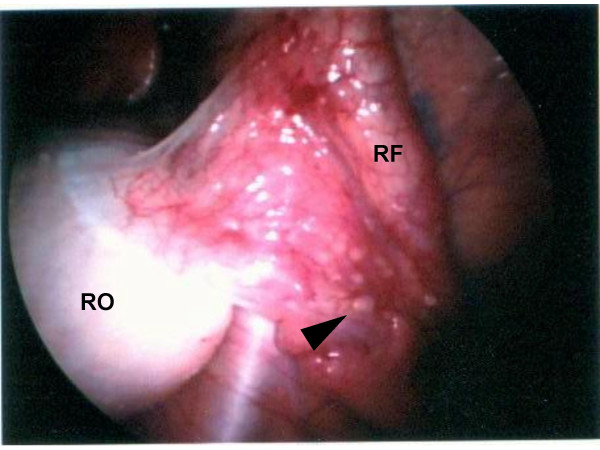
Vesicle-like punctations (arrow) observed on serosa of R fallopian tube (RF). RO = right ovary.

**Figure 4:**
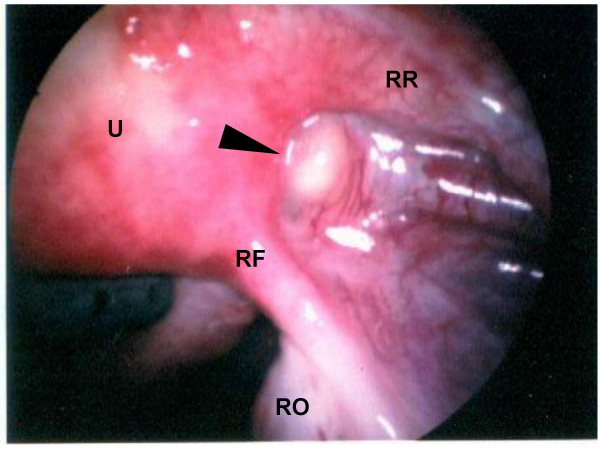
Fibroadipose nodule (arrow) interposed between right round ligament (RR) and right Fallopian tube (RF). U = uterus, RO = right ovary.

Exterior uterine contour appeared normal without evidence of serosal myomas. No gross peritoneal hyperpigmentation, adhesive disease, or puckering was present to suggest endometriosis. However, the surface of the broad ligament demonstrated multiple 1–2 mm vesicle-like punctations [Figure 3] in random distribution which, when biopsied, showed benign peritoneal calcification. Spheroid aggregations of fibroadipose tissue were noted between the round ligament and fallopian tube, especially prominent on the right [Figure 4]. Neither hysteroscopy nor chromopertubation were performed, but normal intrauterine anatomy was determined from pelvic MR.

**Figure 5:**
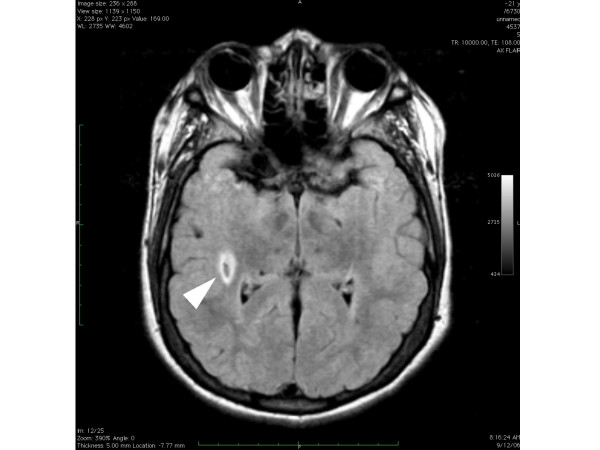
Brain MR demonstrating asymmetric density (arrow) at the inferior margin of L globus pallidus in 46,XX del(4)(q32).

**Figure 6:**
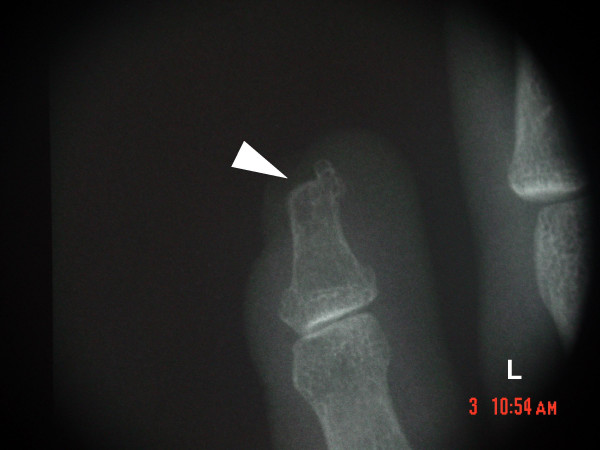
Left hand radiograph showing characteristic abnormal fifth distal phalanx (arrow) in 46,XX del(4)(q32). The defect was bilateral.

Brain MR revealed no gross deviation in normal cortical or ventricular anatomy. However, an area of enhanced signal intensity (approx. 10 mm) was identified at the inferior margin of the right globus pallidus, consistent with old lacunar infarct with surrounding gliosis [Figure 5]. Absence of contrast enhancement at this site suggested a neoplastic process or vascular malformation was unlikely; the benign nature of this lesion was confirmed by head CT with contrast. X-ray of hands demonstrated a bilateral abnormality of the fifth distal phalanges, consistent with developmental dysmorphia or tuft avulsion [Figure 6].

## Discussion

Deletions at 4q have been associated with multiple abnormal physical findings including dysmorphic skull, hypertelorism, cleft palate, short nose with abnormal bridge, and hypoplastic distal phalanx of fifth finger with hooked/volar nail [1,3-5]. The latter of these features has been suggested as virtually pathognomonic for deletion at 4q34 [1]. Interestingly, a small interstitial deletion at 4q31 has been proposed as the "minimal critical region" most likely responsible for the 4q syndrome [6]. The impact of 4q deletion on reproductive and intracranial anatomy remains unknown, however.

That reproductive organs might be adversely impacted by 4q deletion appeared plausible since discontinuous 4q deletions have been implicated in squamous cell and adenocarcinoma of the cervix. Sherwood *et al *concluded that loss at "any chromosome 4 locus occurred in 92% of all (cervical) tumors studied, with the majority of deletions occurring on the long arm of this chromosome" [7]. While further studies to identify specific oncogenes in this chromosomal region continue, for our patient emphasis on tobacco avoidance and routine cervical cancer surveillance via cytology are particularly relevant. While no major deviation from normal was evident regarding uterine and adnexal anatomy, the bilateral fibroadipose aggregates flanking the uterus in the space between the fallopian tube and round ligament represent, to our knowledge, a previously undescribed phenotypic finding for this genetic disorder. Similarly, the observed calcified peritoneal punctations without endometriosis may represent an additional connective tissue derangement of undetermined significance.

The 4q deletion sequence can include mild mental retardation [4,5] and our patient did demonstrate reduced intellectual function and cognitive impairment. While no gross intracranial pathology was identified in this case, high resolution imaging of brain parenchyma did reveal a region of abnormal density, initially thought to be an arteriovenous malformation. Given the well-recognized connective tissue abnormalities seen in 4q deletion, additional imaging modalities were deployed with reassuring results. In the setting of a stable, non-focal neurological examination with no reported seizure activity or head trauma, the intracranial lesion observed in our patient may represent merely an old lacunar infarct rather than a morphological variant directly related to 4q deletion. Unfortunately previous brain imaging studies were not available for comparison, and periodic reassessment was advised.

While the present report describes new morphological features associated with a 4q32 terminal deletion, continued observational studies are needed to show how deletion of genetic material at this locus impacts development.

## Competing interests

The author(s) declare that they have no competing interests.

## Authors' contributions

ESS, MJB, LDP, LPC, LLK, CSD, PRP and JGD all contributed equally to develop and edit the article. ESS was principal physician and coordinated the research and JGD was senior geneticist; PRP directed the genetics laboratory analyses.
